# Effect of Electromagnetic Waves from Mobile Phones on Spermatogenesis in the Era of 4G-LTE

**DOI:** 10.1155/2018/1801798

**Published:** 2018-01-29

**Authors:** Jong Jin Oh, Seok-Soo Byun, Sang Eun Lee, Gheeyoung Choe, Sung Kyu Hong

**Affiliations:** ^1^Department of Urology, Seoul National University College of Medicine, Seoul, Republic of Korea; ^2^Department of Urology, Seoul National University Bundang Hospital, Gyeonggi-do, Republic of Korea; ^3^Department of Pathology, Seoul National University College of Medicine, Seoul, Republic of Korea; ^4^Department of Pathology, Seoul National University Bundang Hospital, Gyeonggi-do, Republic of Korea

## Abstract

**Objective:**

To investigate the effect of long duration exposure to electromagnetic field from mobile phones on spermatogenesis in rats using 4G-LTE.

**Methods:**

Twenty Sprague-Dawley male rats were placed into 4 groups according to the intensity and exposure duration: Group 1 (sham procedure), Group 2 (3 cm distance + 6 h exposure daily), Group 3 (10 cm distance + 18 h exposure daily), and Group 4 (3 cm distance + 18 h exposure daily). After 1 month, we compared sperm parameters and histopathological findings of the testis.

**Results:**

The mean spermatid count (×10^6^/ml) was 398.6 in Group 1, 365.40 in Group 2, 354.60 in Group 3, and 298.60 in Group 4 (*p* = 0.041). In the second review, the mean count of spermatogonia in Group 4 (43.00) was significantly lower than in Group 1 (57.00) and Group 2 (53.40) (*p* < 0.001 and *p* = 0.010, resp.). The sum of the germ cell counts was decreased in Group 4 compared to Groups 1, 2, and 3 (*p* = 0.032). The mean Leydig cell count was significantly decreased in Group 4 (*p* < 0.001).

**Conclusions:**

The longer exposure duration of electromagnetic field decreased the spermatogenesis. Our findings warrant further investigations on the potential effects of EMF from mobile phones on male fertility.

## 1. Introduction

The prevalence of male infertility at the reproductive age has been estimated to be up to 7-8%, and there has been a tendency to increase in recent decades. With the exception of obstructive causes, idiopathic male infertility originates from decreased sperm quality with no organic, genetic, or endocrine alterations in the genital tract [1]. Various environmental factors, such as heat or certain chemical agents, can deteriorate semen quality [2, 3]. With the increasing rate of male infertility, we should focus on the use of mobile phones, which have been increasingly used worldwide. The use of an electromagnetic field (EMF), which is generated from mobile phones, is increasing, which has increased the interest in investigations on its effects on human health [4]. The rapid growth of mobile phone use has been accompanied by a parallel increase in the density of the EMF [5]. The analogue cell phone system introduced in the 1980s operated at an electromagnetic resonance of 902.5 MHz. The recently developed DCS (Digital Cellular System), which uses a radiofrequency of 1,800 MHz, has spread rapidly [6]. More recently, a 4th-generation communication long-term evolution (4G-LTE) system has been used in mobile phones, which can provide a very fast Internet speed over the current radiofrequency rate. 

Public concerns have been raised about the potentially harmful effects of EMF emitted from mobile phones [7]. EMF is considered to have harmful effects on the brain and endocrine system, leading to fatigue, headache, and difficulty in concentrating. Recent cross-sectional studies have highlighted that mobile phone use may be associated with semen quality, and phone use may increasingly contribute to male infertility [1, 8, 9]. A clinical study using mobile phones to evaluate spermatogenesis cannot be performed due to the defined harmful effects; therefore, publications on this topic have been preclinical (animal) or epidemiologic studies of patients visiting infertility clinics. However, these studies showed controversial results on the harmfulness of mobile phone use [10, 11]. EMF rapidly decreases with increasing distance from mobile phones. Therefore, we focus on the distance from the EMF and consider that most people have their mobile phones in their pants' pockets. The problem with this setting is that the mobile phone is always on because of its Internet-based communication. The purpose of this study is to determine the effect of the mobile phone (4G-LTE based) on spermatogenesis in rats according to distance and exposure duration over 2 GHz.

## 2. Materials and Methods

After the approval of the Institutional Animal Care and Use Committee of the Korean Institute of Radiological and Medical Sciences (KIRAMS), our study was performed. And we followed the rules for the care and use of laboratory animals.

### 2.1. Animals

For the spermatogenesis study, 20 male Sprague-Dawley rats (Korea Biolink, Korea) aged 4 weeks were used. Animals were housed in autoclaved cages (polysulfone, 369*L* × 215*W* × 185*H* (mm)) that were maintained at a controlled temperature (22 ± 2°C) and humidity (50 ± 15%) under a regular 12-hour light (150~300 lux) and 12-hour dark cycle for 2 weeks. All animals were maintained at an animal care facility, and food (20% protein, R3+, SAFE Inc., France) and water were supplied ad libitum.

### 2.2. EMF Exposure System

The EMF device and shield room were designed to irradiate the experimental groups of rats in equal levels and densities of EMF by the Institute of Biomedical Engineering. The power was generated by a specific generator (Korea E3 Test Institute) operating at 2.104 GHz continuously. For rats weighing between 150 and 200 g, whole-body averaged specific absorption rate (SAR) was determined to be 3.0 W/kg. This resembles the amount of daily human exposure for the general public. 

To measure SAR, the electromagnetic field values were measured with an electric field probe while the transmitter was on, and then these measured values were used in an electromagnetic field solver to find the electric field distribution in the cage and inside the rat as in previous study settings [12].

### 2.3. Experimental Design

**Figure 1:**
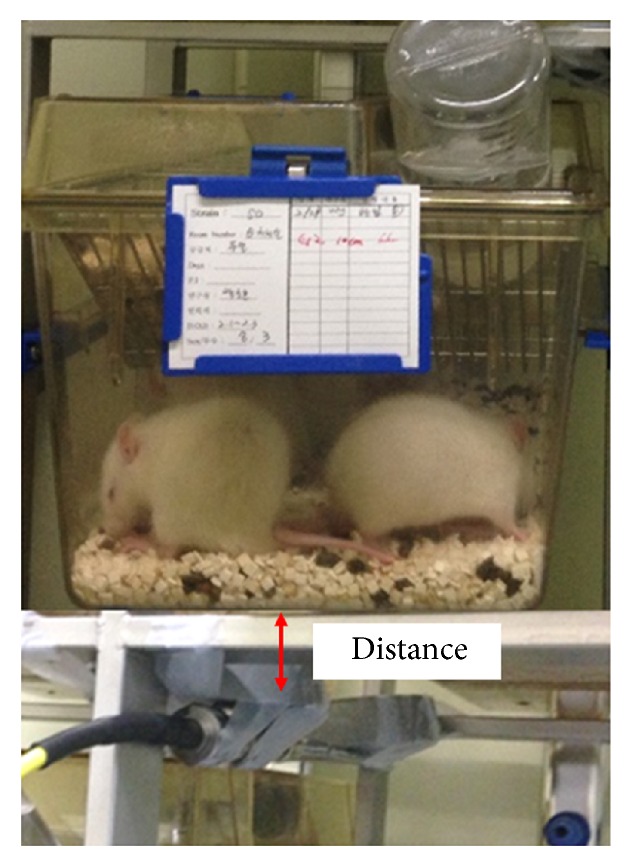
Electromagnetic field exposure device located on the ventral side of the cage.

For the spermatogenesis study, the rats were randomly assigned to four groups according to the exposure time and distance: Group 1 (sham procedure group), Group 2 (3 cm distance + 6 h exposure per day), Group 3 (10 cm distance + 18 h exposure per day), and Group 4 (3 cm distance + 18 h exposure per day). The EMF device located at the bottom of the cage was used as the EMF apparent plate (Figure 1). After 28 days of exposure to EMF, the rats were euthanized with a lethal intraperitoneal dose of pentobarbital, and both testes were excised and weighed. Animal protocols used in the research reported here were approved by the Animal Care Committee of the Special Animal Lab.

### 2.4. Semen Analysis

Semen analysis was performed in two stages. First, semen samples were collected from the left caudal epididymis and were assessed for the number and gross morphology without the investigator knowing which samples were from which group. The sperm was counted under a light microscope with a Neubauer hemocytometer (Paulmarine, Germany) by a veterinarian (first review). The right testis from each rat was histopathologically examined and the number of spermatids per seminiferous tubule was counted by a single uropathologist (second review). The examination was repeated 30 times in different tissues in the right testis. The results of semen analysis and histopathologic spermatid counts were compared among each group.

### 2.5. Testicular Histology

The right testes were removed from three rats of each group and they were bisected and fixed in 10% Bouin solution for 24 h at room temperature. After fixation, five slices of the testis were taken from each group. They were subjected to routine histologic tissue processing including dehydration, clearing, impregnation, and embedding in paraffin. Paraffin blocks were sliced to 3 *μ*m thickness with a microtome and the slices were subjected to routine hematoxylin and eosin (H&E) staining. A single uropathologist who was blinded to the experimental group details evaluated the slides under a light microscope (Olympus, BX52).

The histopathologic analysis included spermatogenic arrest, atrophic seminiferous tubules, thickening of the basal lamina, Leydig cell hyperplasia, degree of interstitial edema, and histologic abnormality in the epididymis. Semiquantitative histopathologic analysis was also performed regarding spermatogenesis. The number of seminiferous tubules per ×100 microscope field, the number of spermatogonia per seminiferous tubule, the number of spermatocytes per seminiferous tubule, the number of spermatids per seminiferous tubule, and the number of Leydig cells per ×400 microscope field were counted in each slide. Additionally, 30 randomly selected seminiferous tubules from each group were evaluated using Johnsen's scoring system [13].

### 2.6. Statistical Analysis

Statistical analysis was performed using one-way ANOVA. A *p* value < 0.05 was considered statistically significant.

## 3. Results

### 3.1. Baseline Results

The baseline change during experiments is shown in Table 1. The final body weight and weight gain were not significantly different among the groups. The rectal temperature was measured before and after. The mean initial temperatures were 36.8 (G1), 37.0 (G2), 36.8 (G3), and 36.7 (G4) for each group (*p* = 0.905). The final rectal temperatures were 36.8 (G1), 36.9 (G2), 36.9 (G3), and 36.9 (G4). There was no significant change or difference among the groups or during the experiments. There was no difference in the food intake among the groups. No abnormal gross findings were observed.

### 3.2. Semen Analysis

**Figure 2:**
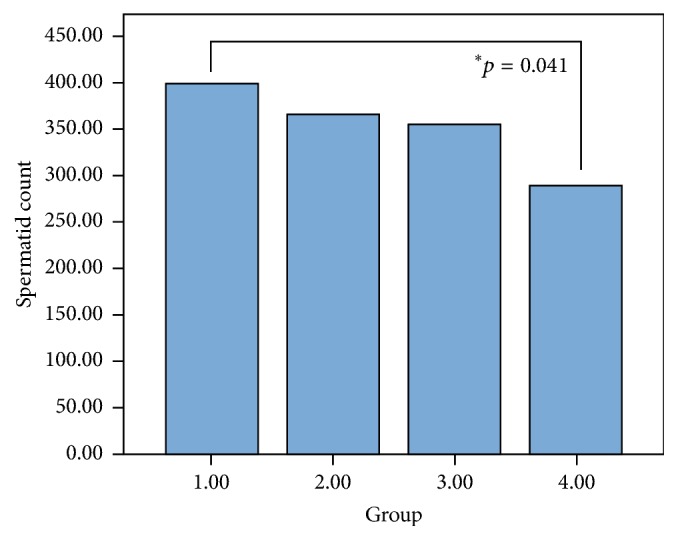
Spermatid count by the hemocytometer of each group. Group 1: sham procedure group; Group 2: 3 cm distance + 6 h exposure per day; Group 3: 10 cm distance + 18 h exposure per day; Group 4: 3 cm distance + 18 h exposure per day.

The semen samples were collected from the left caudal epididymis, as shown in Table 2 (first review by a veterinarian). The mean spermatid count was 398.6 ± 78.52 in Group 1, 365.40 ± 81.25 in Group 2, 354.60 ± 28.62 in Group 3, and 298.60 ± 10.22 in Group 4. There were no significant differences between Groups 1, 2, and 3. Only Group 4 had significantly lower spermatid counts than Group 1 (*p* = 0.041, Figure 2).

### 3.3. Histologic Spermatogenesis

**Figure 3:**
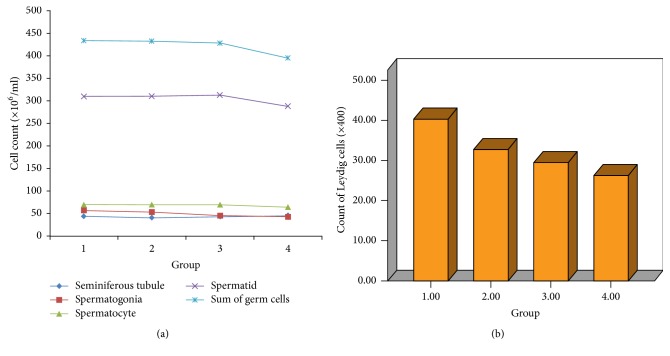
(a) The count of seminiferous tubules, spermatogonia, spermatocytes, and spermatids and the sum of germ cells. (b) The count of Leydig cells according to each group.

The results of the second review in the right testes by a uropathologist are also shown in Table 2. The mean count of spermatogonia in Group 4 (43.00 ± 7.46) was significantly lower than in Group 1 (57.00 ± 15.07) and Group 2 (53.40 ± 12.13) (*p* < 0.001 and *p* = 0.010, resp.) (Figure 3(a)). The mean spermatocyte and spermatid counts were relatively, but not significantly, lower in Group 4 than the others. The sum of germ cell counts was decreased in Group 4 (394.37  ±  47.33) compared to Group 1 (432.80 ± 47.69), Group 2 (432.17 ± 62.78), and Group 3 (427.50 ± 54.96) (*p* for trend =0.032, Figure 3(a)). The mean Leydig cell count was 40.43  ±  13.12 in Group 1, 32.83  ±  7.41 in Group 2, 29.47  ±  5.58 in Group 3, and 26.20 ±  3.96 in Group 4 (*p* for trend <0.001), which showed a gradual decrease with increasing exposure level (Figure 3(b)).

### 3.4. Testicular Morphologic Change

**Figure 4:**
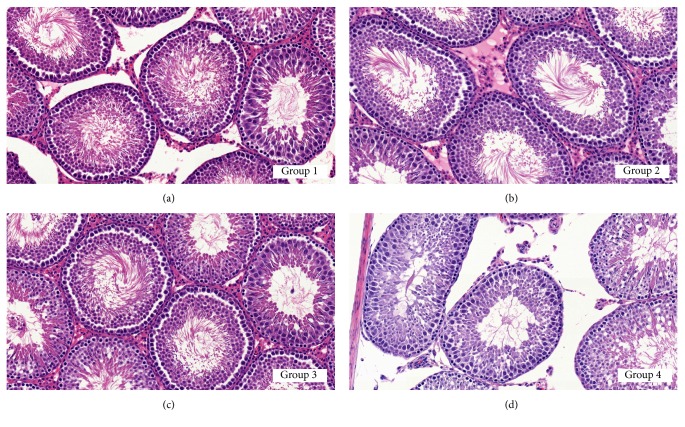
Photomicrographs of testes in each group. Note the decreased elongated spermatids (late spermatids) in Group 4 (d) compared with other groups [(a) Group 1, (b) Group 2, (c) Group 3]. (a–d) H&E, ×400.

The histopathologic analysis detected no morphologic abnormality related to chronic testicular atrophy including spermatogenic arrest, atrophic seminiferous tubules, thickening of the basal lamina, and Leydig cell hyperplasia in all the groups. The degree of interstitial edema was variable area by area; however, no significant difference was noted among the groups. No histologic abnormality was noted in the epididymis of each group. Semiquantitative histopathologic analysis revealed decreased elongated spermatids in Group 4 (Figure 4(d)) compared with the other groups, whereas no morphologic abnormality was noted in germ cells (Figure 4). The mean Johnsen biopsy score was measured to be 9.67 ± 0.55 in Group 1, 9.63 ± 0.56 in Group 2, 9.53 ± 0.57 in Group 3, and 9.23 ± 0.68 in Group 4 (Table 2). The mean Johnsen biopsy score in Group 4 was significantly lower than in Groups 1 and 2 (*p* = 0.027 and *p* = 0.048, resp.).

## 4. Discussion

Mobile phones have become an important part of daily life [14]. The rapid growth of mobile phone use has been accompanied by a parallel increase in the EMF density [5, 15]. The direct biological effects of an electromagnetic field are divided into thermal effects by the electromagnetic field energy absorption, stimulation function by the induced electric current, and athermic action by the long-term exposure.

No clinical study has evaluated the effect of EMF on the reproductive function in humans, but some epidemiologic studies have shown that mobile phone use has a negative effect on spermatogenesis [16]. Agarwal et al. [9] showed, in 360 patients who visited an infertility clinic, that cell phone use decreased semen quality in men by decreasing the sperm count, motility, and viability. Fejes et al. [1] also showed, in their cross-sectional study (infertility clinics), that the duration of cell phone use was negatively correlated with the proportion of rapid progressive motile spermatozoa and motility. However, in these human reports, we could not control the amount and duration of EMF exposure; therefore, inhomogeneous results have been reported. Rago et al. [17] in a recent study reported that none of the conventional sperm parameters were significantly altered. We could presume that there is a hazardous effect of cell phones emitting an EMF on spermatogenesis in the absence of direct clinical evidence.

In an animal study that could control the exposure level to rats or mice, controversial results have been reported. The current study reported in 30 Sprague-Dawley rats with an EMF of 900 MHz (10 min for 30 days) that the sperm count and motility were significantly decreased [18]. Another recent report by Lee et al. [19] showed that 2.45 GHz Wi-Fi significantly decreased the sperm count and motility. However, other studies have not demonstrated a sperm count decrease in the experimental group (EMF exposure) compared to the control group [10, 11, 19, 20]. Still, the aforementioned reports homogenously showed that sperm motility and quality were significantly lower in the EMF-exposed animals than in controls. A meta-analysis by Liu et al. [16] in 2014 included 18 studies on 3,947 men and 186 rats and showed that mobile phone use had no adverse effects on the semen parameters in human studies. However, they concluded that EMF exposure had harmful effects on sperm motility in an animal study. 

EMF exposure could be harmful to spermatogenesis. If the spermatogenesis system is exposed to EMF, vital sperm or germ cells could have decreased motility or vitality. However, there was a compensatory mechanism, and the sperm count or health of the sperm could be increased in that time. In spite of the effect of the long duration of exposure to EMF on the spermatogenesis system, the changes could be reversed. Based on previous studies, we can summarize the results. In a study by Kim et al. [21], the authors used 2.45 GHz EMF for 1-2 h per day. They observed that the Leydig cell count was significantly higher in the 2 h exposed rats than in the sham procedure group. The experimental group had a higher testosterone level because of the higher number of Leydig cells. Additionally, their results showed that the difference in the sperm count between the experimental and control groups was not statistically significant and there were no morphologic changes. In a human study, Rago et al. [17] reported that there was no difference in the sperm-related parameters and testosterone level between non-cell phone users and >4 h users. However, under the circumstances of long-term exposure and short distance from the EMF, the sperm motility decreased, which was observed in all studies. After the Leydig cell counts decreased, the testosterone level also decreased, as can be found in Sepehrimanesh et al.'s study [4]. The authors found that the long-term effect of 900 MHz EMF significantly decreased the Leydig cell count and testosterone level after 30 days. As in this study, we found that the Leydig cell number significantly decreased with increasing EMF exposure level. Additionally, our study had a definite sperm count decrease, which might be from (1) EMF using 4G-LTE above 2.0 GHz, (2) the short distance between the EMF machine and the cage, and (3) a long daily exposure time. Therefore, we think that the reason of the decline of spermatogenesis might be the decrease of Leydig cell function, rather than the thermal effect.

Continuous exposure to EMF induced apoptosis of spermatogenic cells in a duration- and dose-dependent manner [22–24]. In quantitative analysis, there are significantly fewer mature spermatogenic cells (spermatid and spermatozoa) in exposed mice than in controls [22]. In contrast, occupational exposure or short-term exposure to EMF showed no adverse effects on spermatogenesis in some studies [25]. This result demonstrates that cell proliferation and DNA damage induced by ELF-EMF in a specific cell type may be mediated by an increase in nucleotide mismatch. It is suggested that exposure to EMF caused a transient mitogenic effect followed by a DNA-damaging effect [26]. However, with long-term exposure, total germ cell transformation was significantly higher and the cell population in 2 N spermatogonia was significantly lower than in control groups [27]. It has been suggested that long-term exposure to an EMF could affect the proliferation and differentiation of spermatogonia. Therefore, in our study, the number of spermatogonia was decreased with increasing exposure level. The mechanism of the germ cell apoptotic pathway with exposure to EMF is poorly understood. However, on the basis of the serial biological response induced by EMF exposure from each reported result, we can comprehensively speculate on the germ cell apoptotic pathway response to EMF exposure whereby initially mature spermatids degenerate in response to direct cytotoxicity from a high dose of EMF [15].

The SAR value was calculated as proportional to the square of the internal electric field strength; however, permission for SAR was reported in the measurement of the chest, abdomen, or head portion. There was no specific guideline for SAR in reproductive organs. Therefore, we used this SAR guideline in our experiments. Another important factor was the exposure duration. The SAR value is independent of the duration (or repetition) of exposure; therefore, we focus on the long duration of exposure in this analysis. We conclude that a relatively short duration of EMF exposure was relatively safe; however, a long daily duration of cell phone use could be harmful to fertile men. In this study, we could not measure the hormone levels, such as testosterone, FSH, or LH. However, we can predict that the testosterone level will change with the change in the Leydig cell count. In this context, another harmful effect of the EMF, such as the thermal effect, could impact spermatogenesis. However, the rectal temperature of the experimental group was not significantly different from that of the control group.

## 5. Conclusions

In this study, we observed that a long duration of 4G-LTE-based EMF had a harmful effect on spermatogenesis. In particular, the sperm and Leydig cell counts significantly decreased in the long duration exposure group, showing that continuous cell phone use could be hazardous for fertile men, especially adolescent men. However, the 10 cm distance group with the same duration and same energy was relatively less affected by EMF, which could indicate that carrying a cell phone in the pants' pocket (continuous access mode) could be harmful.

## Figures and Tables

**Table 1:** Baseline characteristics.

	Group 1 (Sham procedure)	Group 2 (3 cm, 6 h/d)	Group 3 (10 cm, 18 h/d)	Group 4 (3 cm, 18 h/d)	*p*-value
Body weight, initial (mean ± SD), gm	117.4 ± 5.73	117.2 ± 3.63	118.0 ± 4.64	118.4 ± 7.20	0.984
Body weight, final (mean ± SD), gm	270.8 ± 18.67	268.2 ± 23.9	264.4 ± 9.29	256.6 ± 10.43	0.573
Weight gain (mean ± SD), gm	153.4 ± 18.2	151.0 ± 22.0	146.4 ± 9.6	138.2 ± 12.9	0.494
Rectal temperature, initial (mean ± SD), °C	36.8 ± 0.34	37.0 ± 0.24	36.8 ± 0.35	36.7 ± 0.22	0.905
Rectal temperature, final (mean ± SD), °C	36.8 ± 0.29	36.9 ± 0.18	36.9 ± 0.08	36.9 ± 0.13	0.970
Food intake (mean), gm	23.3	23.0	22.5	21.5	0.857
Testis weight, gm	1.85 ± 0.21	1.79 ± 0.34	1.84 ± 0.84	1.91 ± 0.11	0.871

Group 1—Sham procedure group; Group 2—3 cm distance + 6 h exposure daily; Group 3—10 cm distance + 18 h exposure daily; and Group 4—3 cm distance + 18 h exposure daily.

**Table 2:** Semen parameters according to electromagnetic field exposure level.

	Group 1 (Sham procedure)	Group 2 (3 cm, 6 h/d)	Group 3 (10 cm, 18 h/d)	Group 4 (3 cm, 18 h/d)	*p*-value
Hemocytometer view^*∗*^					
Spermatid count (mean ± SD), ×10^6^	398.6 ± 78.52	365.40 ± 81.25	354.60 ± 28.62	298.60 ± 10.25	0.057
Histopathologic view^*∗∗*^ (mean ± SD)					
Count of Seminiferous tubule, ×100	43.70 ± 4.09	40.77 ± 5.14	42.53 ± 5.76	45.00 ± 6.20	0.021
Spermatogonia	57.00 ± 15.07	53.40 ± 12.13	45.23 ± 14.71	43.00 ± 7.46	<0.001
Spermatocyte	69.73 ± 12.25	69.30 ± 12.16	69.43 ± 11.60	63.97 ± 7.81	0.189
Spermatid	309.07 ± 44.99	309.47 ± 52.13	312.83 ± 54.52	287.40 ± 48.50	0.102
Sum of germ cell	432.80 ± 47.69	432.17 ± 62.78	427.50 ± 54.96	394.37 ± 47.33	0.032
Leydig cell, ×400	40.43 ± 13.12	32.83 ± 7.41	29.47 ± 5.58	26.20 ± 3.96	<0.001
Johnsen's biopsy score	9.67 ± 0.55	9.63 ± 0.56	9.53 ± 0.57	9.23 ± 0.68	0.022

^*∗*^Hemocytometer view was calculated by hemocytometer in specimen which obtained by semen samples were collected from the left caudal epididymis;  ^*∗∗*^Histopathologic view was calculated by light microscope after H-E stain by uro-pathologist.

## References

[1] Fejes I., Závaczki Z., Szöllosi J. (2005). Is there a relationship between cell phone use and semen quality?. *Journal of Reproductive Systems*.

[2] Chowdhury A. K., Steinberger E. (1964). A quantitative study of the effect of heat on germinal epithelium of rat testes. *American Journal of Anatomy*.

[3] Jégou B., Laws A. O., de Kretser D. M. (1984). Changes in testicular function induced by short‐term exposure of the rat testis to heat: further evidence for interaction of germ cells, Sertoli cells and Leydig cells. *International Journal of Andrology*.

[4] Sepehrimanesh M., Saeb M., Nazifi S., Kazemipour N., Jelodar G., Saeb S. (2014). Impact of 900 MHz electromagnetic field exposure on main male reproductive hormone levels: a Rattus norvegicus model. *International Journal of Biometerology*.

[5] Kesari K. K., Kumar S., Nirala J., Siddiqui M. H., Behari J. (2013). Biophysical evaluation of radiofrequency electromagnetic field effects on male reproductive pattern. *Cell Biochemistry and Biophysics*.

[6] Roelandts R. (2003). Cellular phones and the skin. *Dermatology*.

[7] Agarwal A., Singh A., Hamada A., Kesari K. (2011). Cell phones and male infertility: a review of recent innovations in technology and consequences. *The International Brazilian Journal of Urology*.

[8] Gorpinchenko I., Nikitin O., Banyra O., Shulyak A. (2014). The influence of direct mobile phone radiation on sperm quality. *Central European Journal of Urology*.

[9] Agarwal A., Deepinder F., Sharma R. K., Ranga G., Li J. (2008). Effect of cell phone usage on semen analysis in men attending infertility clinic: an observational study. *Fertility and Sterility*.

[10] Dasdag S., Akdag M. Z., Aksen F. (2003). Whole body exposure of rats to microwaves emitted from a cell phone does not affect the testes. *Bioelectromagnetics*.

[11] Yan J.-G., Agresti M., Bruce T., Yan Y. H., Granlund A., Matloub H. S. (2007). Effects of cellular phone emissions on sperm motility in rats. *Fertility and Sterility*.

[12] Dasdag S., Taş M., Akdag M. Z., Yegin K. (2015). Effect of long-term exposure of 2.4 GHz radiofrequency radiation emitted from Wi-Fi equipment on testes functions. *Electromagnetic Biology and Medicine*.

[13] Johnsen S. E. (1970). Testicular biopsy score count-a method for registration of spermatogenesis in human testes: normal values and results in 335 hypogonadal males. *Hormones*.

[14] Merhi Z. O. (2012). Challenging cell phone impact on reproduction: a review. *Journal of Assisted Reproduction and Genetics*.

[15] Lee S.-K., Park S., Gimm Y.-M., Kim Y.-W. (2014). Extremely low frequency magnetic fields induce spermatogenic germ cell apoptosis: Possible mechanism. *BioMed Research International*.

[16] Liu K., Li Y., Zhang G. (2014). Association between mobile phone use and semen quality: A systemic review and meta-analysis. *Andrology*.

[17] Rago R., Salacone P., Caponecchia L. (2013). The semen quality of the mobile phone users. *Journal of endocrinological investigation*.

[18] Guan M., Tang W., Hang J. (2012). Effects of mobile phone radiation on semen quality of rat. *Chinese Journal of Andrology*.

[19] Lee H.-J., Pack J.-K., Kim T.-H. (2010). The lack of histological changes of CDMA cellular phone-based radio frequency on rat testis. *Bioelectromagnetics*.

[20] Ribeiro E. P., Rhoden E. L., Horn M. M., Rhoden C., Lima L. P., Toniolo L. (2007). Effects of subchronic exposure to radio frequency from a conventional cellular telephone on testicular function in adult rats. *The Journal of Urology*.

[21] Kim J. Y., Kim H. T., Moon K. H., Shin H. J. (2007). Long-term exposure of rats to a 2.45 GHz electromagnetic field: effects on reproductive function. *Korean Journal of Urology*.

[22] Lee J. S., Ahn S. S., Jung K. C., Kim Y.-W., Lee S. K. (2004). Effects of 60 Hz electromagnetic field exposure on testicular germ cell apoptosis in mice. *Asian Journal of Andrology*.

[23] Kim Y.-W., Kim H.-S., Lee J.-S. (2009). Effects of 60 Hz 14 *μ*T magnetic field on the apoptosis of testicular germ cell in mice. *Bioelectromagnetics*.

[24] Kim H.-S., Park B.-J., Jang H.-J. (2014). Continuous exposure to 60Hz magnetic fields induces duration- and dose-dependent apoptosis of testicular germ cells. *Bioelectromagnetics*.

[25] Duan W., Liu C., Wu H. (2014). Effects of exposure to extremely low frequency magnetic fields on spermatogenesis in adult rats. *Bioelectromagnetics*.

[26] Wolf F. I., Torsello A., Tedesco B. (2005). 50-Hz extremely low frequency electromagnetic fields enhance cell proliferation and DNA damage: Possible involvement of a redox mechanism. *Biochimica et Biophysica Acta (BBA) - Molecular Cell Research*.

[27] Furuya H., Aikawa H., Hagino T., Yoshida T., Sakabe K. (1998). Flow Cytometric Analysis of the Effects of 50Hz Magnetic Fields on Mouse Spermatogenesis. *Japanese Journal of Hygiene*.

